# Influence of density and salinity on larval development of salt‐adapted and salt‐naïve frog populations

**DOI:** 10.1002/ece3.6069

**Published:** 2020-02-05

**Authors:** Molly A. Albecker, Matthew Pahl, Melanie Smith, Jefferson G. Wilson, Michael W. McCoy

**Affiliations:** ^1^ Department of Biology Northeastern University Marine Science Center Northeastern University Nahant MA USA; ^2^ Department of Biology Howell Science Complex East Carolina University Greenville NC USA

**Keywords:** anuran amphibian, complex life cycles, density dependence, local adaptation, salt tolerance

## Abstract

Environmental change and habitat fragmentation will affect population densities for many species. For those species that have locally adapted to persist in changed or stressful habitats, it is uncertain how density dependence will affect adaptive responses. Anurans (frogs and toads) are typically freshwater organisms, but some coastal populations of green treefrogs (*Hyla cinerea*) have adapted to brackish, coastal wetlands. Tadpoles from coastal populations metamorphose sooner and demonstrate faster growth rates than inland populations when reared solitarily. Although saltwater exposure has adaptively reduced the duration of the larval period for coastal populations, increases in densities during larval development typically increase time to metamorphosis and reduce rates of growth and survival. We test how combined stressors of density and salinity affect larval development between salt‐adapted (“coastal”) and nonsalt‐adapted (“inland”) populations by measuring various developmental and metamorphic phenotypes. We found that increased tadpole density strongly affected coastal and inland tadpole populations similarly. In high‐density treatments, both coastal and inland populations had reduced growth rates, greater exponential decay of growth, a smaller size at metamorphosis, took longer to reach metamorphosis, and had lower survivorship at metamorphosis. Salinity only exaggerated the effects of density on the time to reach metamorphosis and exponential decay of growth. Location of origin affected length at metamorphosis, with coastal tadpoles metamorphosing slightly longer than inland tadpoles across densities and salinities. These findings confirm that density has a strong and central influence on larval development even across divergent populations and habitat types and may mitigate the expression (and therefore detection) of locally adapted phenotypes.

## Graphical abstract

We compare how the dual impact of density and salinity affect larval development in salt‐adapted and non‐salt‐adapted amphibian populations. We find that density is the predominate factor governing development and metamorphosis across divergent populations. These findings confirm that density has a strong and central influence on larval development even across divergent populations and habitat types and may mitigate the expression (and therefore detection) of locally adapted phenotypes.

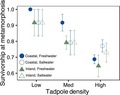

## 1 INTRODUCTION

Habitat quality is in a state of flux worldwide due to climate change, urbanization, and other anthropogenic disturbances (Grimm et al., 2013; Pereira et al., 2010). Habitats are also becoming increasingly fragmented into collages of favorable and unfavorable habitat patches (Haddad et al., 2015; Lewis, 2006). Concomitant changes in the quality and contiguity of habitats increase the likelihood that populations of affected species will inhabit unfavorable patches that alter population growth and dispersal rates. These effects may be particularly impactful for species that use spatially circumscribed habitat patches (e.g., ponds) or that occur in spatially structured population networks (Cushman, 2006; Keinath et al., 2017).

Populations that reside in habitats that become abiotically unsuitable will typically decline to extirpation (Grimm et al., 2013; Stuart et al., 2004), unless they are able to locally adapt to the altered environmental conditions via evolutionary rescue (Bell, 2017; Gomulkiewicz & Holt, 1995; Holt, 2011). Evolutionary rescue is typified by a triphasic process that leads to a U‐shaped pattern in population abundances through time. The first phase is marked by a decline in population abundance following an environmental change. The second phase encapsulates a period during which the population stops declining, but levels off at population abundances that are low enough to be vulnerable to stochastic extinction events. The third phase occurs as the equilibrium abundances begin to increase above the stochastic extinction thresholds as a result of an increase in the frequency of adaptive alleles in the population (Carlson, Cunningham, & Westley, 2014).

Adaptive alleles that yield positive population growth may manifest into innumerable phenotypic forms (e.g., behavior, morphology, and development) that improve fitness. However, if the adaptive phenotype is affected by changes in density, the ability for individuals to realize the benefits of the adaptive responses that emerge when the populations are at low densities may dissipate as population sizes increase and density‐dependent processes dominate. For example, faster growth rates may improve fitness, but the presence of competitors may usurp resources necessary to sustain accelerated growth rates. Therefore, the adaptive value of increased growth rates may only be realized during stages one and two of evolutionary rescue when the population is declining or being maintained at low densities, but may be masked during stage three by negative density dependence as the population recovers. Understanding how density‐dependent processes mitigate the performance of adaptive phenotypes is needed to improve our ability to predict impacts of local adaptation on long‐term population dynamics (Urban et al., 2016).

For organisms with complex life cycles, a common adaptation to changes in environmental quality is to alter growth or developmental rates of the larval stage (Richter‐Boix, Tejedo, & Rezende, 2011). For example, larval amphibians in high‐risk habitats characterized by abundant predators or rapid desiccation often increase developmental rates to reach metamorphosis quickly and avoid certain mortality in the deteriorating larval environment (Werner & Gilliam, 1984; Wilbur & Collins, 1973). Spadefoot toads (Genus *Scaphiopus*) are perhaps the most exemplary of this strategy as several species have evolved very short larval periods to deal with exceedingly ephemeral ponds within desert environments (Bragg, 1965; Denver, 1997; Levis & Pfennig, 2018; Newman, 1992). Many other species can also speed up development or growth, but this trait is often plastic and can be moderated according to perceived risk (Abrams & Matsuda, 1996; McCoy, 2007; Peacor & Werner, 2004; Richter‐Boix et al., 2011).

Although speeding up development may be adaptive, individual growth rates are also affected by a wide variety of other biotic and abiotic factors like density or exposure to saltwater. For example, individual growth rates have been shown to be reduced via negative density dependence even in the face of other environmental pressures that are known to promote rapid metamorphosis (Richards, 1958; Wells, 2007). Reductions in individual growth rates with increases in conspecific density can stem from resource limitation, intraspecific competitive interactions (Griffiths, 1991), accumulation of nitrogenous waste or other chemicals (Rose & Rose, 1965; Smith, 1999), increased physical contact between individuals (Rot‐Nikcevic, Denver, & Wassersug, 2005), and reduced area for swimming (John & Fenster, 1975; John & Fusaro, 1981). In addition to density, salt water can affect growth rates. Salt water is lethal to amphibians in high concentrations (Hopkins & Brodie, 2015), but at sublethal concentrations, salt water reduces tadpole size at metamorphosis and growth rates (Albecker & McCoy, 2019; Christy & Dickman, 2002; Rios‐López, 2008; Wu & Kam, 2009). Some species may be able to compensate for reduced growth if released from salt stress (Squires, Bailey, Reina, & Wong, 2010), but others cannot (Wu, Gomez‐Mestre, & Kam, 2012). Reduced growth in saltwater stems from the allocation of energy toward maintaining internal ion and water balance which reduces the amount of energy available for somatic growth (Bernabò, Bonacci, Coscarelli, Tripepi, & Brunelli, 2013; Lai, Kam, Lin, & Wu, 2019; Wu, Yang, Lee, Gomez‐Mestre, & Kam, 2014). The effects of salt are variable depending on species, population, and life stage (Albecker & McCoy, 2017; Kearney, Byrne, & Reina, 2016).

In a previous study, Albecker and McCoy (2019) showed that coastal populations of the American green treefrog (*Hyla cinerea*) persist in brackish marshes with salinities above normal amphibian tolerance levels and exhibit a range of adaptive phenotypes in response to salinity that are not present in inland populations (Albecker & McCoy, 2017). These locally adapted coastal populations have higher survival, accelerated larval growth rates, and shorter larval periods than freshwater populations regardless of salinity. This faster paced lifestyle is likely an adaptation to minimize larval mortality risk in highly variable and unpredictable saline environments, since accelerated developmental and growth rates are commonly observed in North American frog species adapted to high‐risk environments (e.g., *Scaphiopus spp*.). However, the negative effects of density may dampen the ability for individuals from locally adapted populations to accelerate life history traits—especially when combined with abiotic stressors like salinity (Jones et al., 2017; Woolrich‐Piña, Smith, Benítez‐Tadeo, Lemos‐Espinal, & Morales‐Garza, 2017; Woolrich‐Piña, Smith, & Lemos‐Espinal, 2015). To our knowledge, previous studies have not compared whether the impacts of density differ between locally adapted and nonlocally adapted populations in a stressful environment. Understanding whether the strength or form of density dependence is different across environmental gradients for locally adapted and nonadapted populations could have important consequences for predicting evolutionary rescue and population dynamics of populations at the frontlines of environmental change. In this study, we test the dual influences of increased density and salinity on the growth, metamorphosis, and survival of *H. cinerea* from salt‐adapted (coastal) and nonsalt‐adapted (inland) populations. We expect that density will strongly influence the expression of adaptive developmental traits (such as growth rates) across both coastal and inland populations, but the effects of increased salinities and density will have a greater effect on inland populations.

## 2 MATERIALS AND METHODS

We collected eight pairs of breeding adult frogs in amplexus from two discrete salt‐adapted (hereafter called coastal) and two discrete salt‐naïve (hereafter called inland) populations of *H. cinerea*. We collected coastal frogs from wetlands near Nags Head along the outer banks' barrier islands of North Carolina on 5 July 2017 and inland frogs were collected from freshwater ponds near Greenville, NC on 6 July 2017. Inland wetlands had surface salinities of 0.23 parts per thousand (ppt) and 0.42 ppt, and coastal wetlands had surface salinities of 1.7 and 1.3 ppt. Salinities were measured using YSI Professional Plus Multiparameter Meter (Xylem, Inc.).

Upon capture, amplexed pairs were placed into covered 5.7‐liter (L) Sterilite^®^ plastic bins containing 2 L of fresh water. The fresh water used in this experiment was dechlorinated tap water treated with API^®^ Tap Water Conditioner, Chalfont, PA. Tap water naturally contains small quantities of dissolved salts, so the salinity of the tap water typically measures around 0.5 ppt (Albecker, personal observation). Pairs were left in situ at the edge of the breeding ponds overnight to lay eggs. Adult frogs were released the following morning at the site of collection, and their eggs were transported to the laboratory and maintained at 25°C in natural light. Each container was aerated to prevent stagnation while the embryos developed and hatched. Hatching occurred after approximately 48 hr. Following the transition from dependence on yolk reserves to active foraging (Gosner stage 25) (Gosner, 1960), hatchlings from each geographic location (e.g., coastal and inland) were combined and maintained in 15 L of aerated, conditioned tap water. Six replicate subsamples of 2, 4, and 8 individuals were haphazardly sampled from the coastal and inland cohorts. Each subsampled group was immediately placed into an 11 × 13‐centimeter (diameter × height) plastic container with either 400 ml of fresh water or 4 ppt saltwater. The three density treatments translate into densities of 22 tadpoles/m^2^, 44/m^2^, and 88/m^2^, respectively, which are within the range of densities reported for natural populations of many pond breeding species (Alford, 1986). These densities will be referred to as low (2‐individuals), medium (4‐individuals), and high (8‐individuals) density treatments for the remainder of the paper. Saltwater was made by mixing Instant Ocean Sea Salt^®^ into treated fresh water which is a NaCl‐based aquarium salt that mimics the chemical composition of natural seawater.

The 12 experimental treatments were arranged in six replicate spatial blocks (*N* = 72 total experimental units; 3 densities × 2 salinities × 2 locations × 6 replicates). Temperature was maintained at 27°C, with a 12 hr light/dark cycle. Mortality was recorded daily, and deceased individuals were immediately removed. Full water changes occurred every second day maintaining the same salinity, and tadpoles were fed 75 mg of spirulina flakes (O.S.I.^®^) after every water change (Berven & Chadra, 1988). Once per week, tadpoles were weighed and measured (snout‐vent length and total length) using Neiko^®^ digital calipers and a GeneMate^®^ digital balance. Upon reaching stage 42, defined as the point that forelimbs emerge (Gosner, 1960), metamorphs were weighed, measured, and placed into individual covered containers (same dimensions as the larval containers) lined with a moist paper towel to complete metamorphosis. Animals were collected under NC wildlife collection license (17‐SC00840), and all experimental protocols were approved by ECU's Animal Care and Use Committee (IACUC #D314).

### 2.1 Statistical methods

We tested for the effects of salinity, density, and location on tadpole survival, tadpole growth, size at metamorphosis, and age at metamorphosis using generalized linear mixed effects models using package “lme4” (Bates, Martin, Bolker, & Walker, 2015) in the R statistical programing environment version 3.5.0 (Team, 2018). For all endpoints except growth, we treated salinity, density, and location as fixed effects, and replicate as a random effect. For each analysis, we started with the full interaction model, which included the fully crossed effects of location, salinity, and density, and then reduced model complexity by sequentially dropping higher order terms based on likelihood ratio tests.

For our analysis of survival, we assumed a binomial error distribution, and we assumed a Poisson error distribution to analyze the number of days between hatching and metamorphosis (i.e., age at metamorphosis defined as Gosner stage 42, the day of forelimb emergence; Gosner, 1960). We use Poisson because these data are integers derived from a count‐based sampling effort which is assumed to conform to the Poisson distribution (Bolker et al., 2009). To test for differences in size at metamorphosis, we analyzed data on total length at developmental stage 42 assuming a log‐normal error distribution. We use log‐normal distribution for size measurements because size is by definition bound at zero, and model diagnostics improved following log‐transformation.

### 2.2 Growth

We analyze change in total length (snout to tail) through time (Y(t)) by fitting a Gompertz growth model using the method of maximum likelihood in the R package “bblme” (Bolker & Team, 2017). Specifically, we fit a growth model of the form:

(1) $$Y\left(t\right)=S_{0}^{\left(\frac{\gamma }{\alpha }\right)\left(1-e^{\left(-\alpha t\right)}\right)}$$

where *S*_0_ is an estimate of initial size, *γ* (gamma) corresponds to the maximum size‐specific growth rate, and *α* (alpha) is the exponential decay of size‐specific growth rate, which biologically corresponds with a slowed rate of cell division, cell death, or the suspension of growth as cell differentiation occurs (Harris, 1999). This functional form is consistent with most empirical observations of amphibian growth (Hota, 1994) and permits us to estimate biologically meaningful parameters that provide insights into processes that may underlie changes in growth through time (Harris, 1999). In this species, tail length grows allometrically with body length even in higher salinities, so we use total length as our size metric (Albecker & McCoy, 2019).

To compare growth across treatments, we compare fits of 17 different parameterizations of the Gompertz model to the cup averaged total lengths of tadpoles within each experimental unit. All parameterizations included a single estimate for initial size (*S*_0_) because neither the larval environment or location were expected to affect the size of hatchlings (Albecker & McCoy, 2019). The 17 models included different variants of flexible parameterizations ranging from the most complex scenario that allowed α and *γ* to be estimated as a linear function that included a 3‐way interaction among the treatments (salinity, density, and location) to the simplest scenario that assumed pooled data and ignored treatment effects (i.e., a single estimate of *S*_0,_ α, and *γ* across all treatments). All 17 parameterizations of the models are listed in Table 1. We determined the relative support for candidate models using sample size‐corrected Akaike information criterion (AIC) (Bolker & Team, 2017; Burnham & Anderson, 2004) and infer treatment effects based on the parameterization and estimates associated with the most parsimonious model.

**Table 1:** Models and results used in sample size‐corrected Akaike information criterion (AIC) model comparisons to determine best fit for tests on tadpole total length (in mm) based on the Gompertz growth equation. All 16 combinations of interactive and additive relationships plus the no effects model are shown

S_o_	*γ*	*α*	Rank	dAICc	*df*	Weight
1	Location + Salinity + Density	Location + Salinity * Density	1	0	14	0.31
1	Location * Salinity + Density	Location + Salinity * Density	2	0.2	15	0.28
1	Location + Salinity * Density	Location + Salinity + Density	3	0.9	14	0.20
1	Location + Salinity * Density	Location * Salinity + Density	4	1.4	15	0.16
1	Location + Salinity * Density	Location + Salinity * Density	5	5.0	16	0.03
1	Location + Salinity + Density	Location * Salinity * Density	6	7.3	19	0.01
1	Location * Salinity + Density	Location * Salinity * Density	7	7.7	20	0.006
1	Location * Salinity * Density	Location + Salinity + Density	8	7.8	19	0.006
1	Location + Salinity * Density	Location * Salinity * Density	9	8.8	21	0.004
1	Location * Salinity * Density	Location * Salinity * Density	10	9.7	21	0.002
1	Location * Salinity * Density	Location * Salinity + Density	11	10.0	20	0.002
1	Location + Salinity + Density	Location + Salinity + Density	12	15.8	12	<0.001
1	Location * Salinity + Density	Location + Salinity + Density	13	16.7	13	<0.001
1	Location + Salinity + Density	Location + Salinity + Density	14	16.9	13	<0.001
1	Location * Salinity + Density	Location * Salinity + Density	15	18.7	14	<0.001
1	Location * Salinity * Density	Location * Salinity * Density	16	64.0	28	<0.001
1	1	1	17	610.6	4	<0.001

## 3 RESULTS

### 3.1 Survival

**Figure 1:**
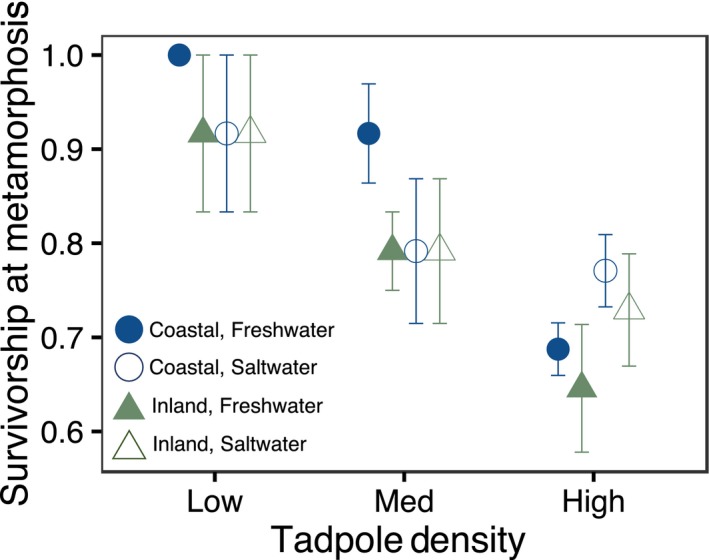
Survival to metamorphosis according to density, salinity, and location. Points represent average survival with standard error. Green triangles represent inland populations; blue circles represent coastal populations. Filled shapes represent tadpoles reared in fresh water, and open shapes represent tadpoles reared in saltwater (4 ppt)

Salinity and location had no effect on survival to metamorphosis (salinity: $X_{6}^{2}$ = 0.29, *p* = .59; location: $X_{6}^{2}$= 1.14, *p* = .29), but density significantly affected survival ($X_{6}^{2}$ = 15.47, *p* = .0004) (Figure 1). Survival declined by approximately 26% in the high‐density treatments relative to the low‐density treatments.

### 3.2 Time to metamorphosis

**Figure 2:**
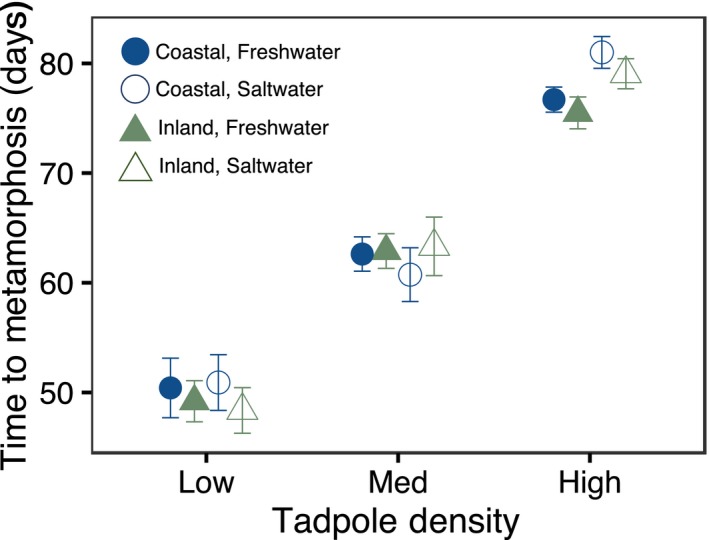
Time to metamorphosis in days according to density, salinity, and location. Points represent average number of days to reach metamorphosis with standard error. Green triangles represent inland populations; blue circles represent coastal populations. Filled shapes represent tadpoles reared in fresh water and open shapes represent tadpoles reared in saltwater (4 ppt)

Location had no effect on the time to metamorphosis ($X_{6}^{2}$ = 0.60, *p* = .44). Salinity had a marginal effect ($X_{6}^{2}$ = 3.09, *p* = .07), and density had a significant effect on the time to metamorphosis ($X_{6}^{2}$ = 466.76, *p* < .0001) (Figure 2). Tadpoles in the low‐density treatment reached metamorphosis the soonest, while tadpoles took the longest time to reach metamorphosis in the high‐density treatment, with individuals in the 4 ppt treatment taking approximately 4 days longer to metamorphose than freshwater individuals.

### 3.3 Length at metamorphosis

**Figure 3:**
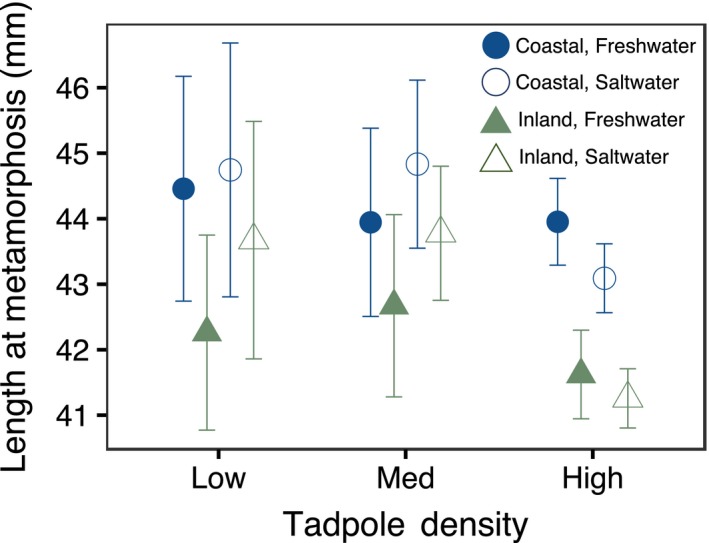
Total length (head to tail) at metamorphosis according to density, salinity, and location. Points represent average length at metamorphosis with standard error. Green triangles represent inland populations; blue circles represent coastal populations. Filled shapes represent tadpoles reared in fresh water and open shapes represent tadpoles reared in saltwater (4 ppt)

Salinity did not affect length at metamorphosis ($X_{6}^{2}$ = 0.11, *p* = .74), but we observed an effect of location ($X_{6}^{2}$ = 8.67, *p* = .003) and a marginal effect of density ($X_{6}^{2}$ = 3.51, *p* = .06) (Figure 3). On average, coastal tadpoles metamorphosed approximately 1.7mm longer than inland tadpoles, and tadpoles that metamorphosed from the low‐density treatments were slightly larger than the high‐density individuals.

### 3.4 Growth rates

**Figure 4:**
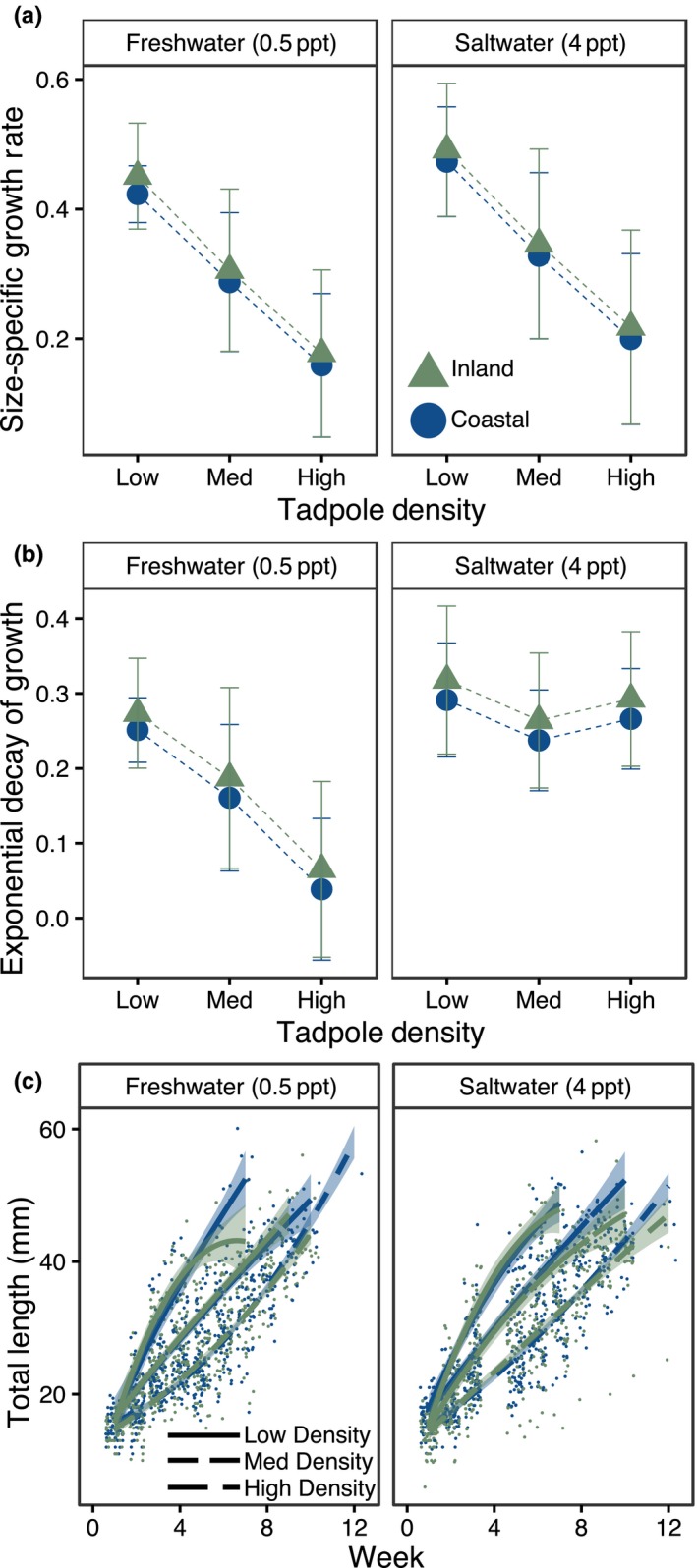
Panels representing tadpole growth according to density, location, and salinity. Panel a is the coefficient estimate for size‐specific growth rate (*γ*), panel b is the coefficient for exponential decay of growth (*α*), and panel c show the model‐fitted curves describing growth through time overlaid on individual measurements. Location is indicated by color, with blue circles representing coastal populations and green triangles representing inland populations. In panel c, the solid line represents the low‐density treatment, the long‐dashed line represents the medium‐density treatment, and the short‐dashed line represents the high‐density treatment. Each panel is divided into the two salinity environments

The Gompertz model (Equation 1) that best fit tadpole growth data had an additive relationship between salinity, density, and location for the *γ* parameter (Table 1), which corresponds to the maximum size‐specific growth rate (Figure 4). The *α* parameter (e.g., exponential decay of growth) had an additive relationship between location and salinity, but an interaction with density. The highest size‐specific growth rates (*γ*) were observed in the low‐density treatments. Growth rate declined in the medium‐density treatments and was the lowest in the high‐density treatments. The exponential decay of growth parameter (*α*) behaved differently. Exponential decay of growth occurred earliest (and thus had the highest parameter estimates) in the low‐density treatments. In the medium‐density treatments, exponential decay had different effects due to salinity in which the growth of tadpoles in the saltwater treatments leveled off sooner, while the growth of the tadpoles in the freshwater treatments leveled off later. Salinity continued to drive divergence in exponential growth decay in the high‐density treatments with saltwater treatments again exhibiting early growth asymptosis, and freshwater treatments exhibiting later asymptosis.

## 4 DISCUSSION

It is well established that the growth and development of amphibian larvae are strongly affected by increases in both salinity (Christy & Dickman, 2002; Rios‐López, 2008; Wu & Kam, 2009) and density (Durnin & Smith, 2001; Golay & Durrer, 1994; Griffiths, 1991; Gromko, Mason, & Smith‐Gill, 1973; John & Fenster, 1975; John & Fusaro, 1981; Rose & Rose, 1965; Rot‐Nikcevic et al., 2005; Smith, 1999). However, we know little about how populations that are locally adapted to better tolerate an abiotic stressor (like salinity) will respond to increases in both salinity and density. We expected that higher densities would exacerbate the impacts of higher salinities for inland (nonsalt‐adapted) populations. But contrary to expectation, we found that tadpole density drove differences in development and metamorphosis for both coastal and inland populations across all endpoints, whereas salinity only affected time to metamorphosis. In the high‐density treatment, both coastal and inland tadpoles from freshwater and saltwater treatments had approximately 25% lower survival, 2.86‐fold slower growth rates, and were 66% older at metamorphosis relative to the low‐density treatments. These findings demonstrate that the form and strength of density dependence are conserved across divergent amphibian populations. These findings are also consistent with the strong negative effects of crowding on population demographics and life history strategy that have been identified in other studies (Brook & Bradshaw, 2006; McCoy, 2007; Vonesh & De la Cruz, 2002), and suggest that biotic factors have a strong and central influence in larval development across divergent populations and habitat types.

Previous work demonstrated that coastal tadpoles have faster growth rates and metamorphosed younger than inland frogs when reared solitarily (Albecker & McCoy, 2019), but in this study, we find that differences in larval growth rates and time to metamorphosis between coastal and inland populations were eliminated. It is possible that increases in density may nullify locally adapted traits in coastal populations. However additional research is needed to link the findings of these studies. Nevertheless, the potential for density dependence to moderate the expression of adaptive traits may have important implications for understanding long‐term outcomes of adaptation to environmental change (Runquist et al., 2019). When populations undergo evolutionary rescue and evolve adaptive traits that permit positive population growth, rebounds in equilibrium abundance are also expected (Bell, 2017). But if the adaptive traits responsible for population recovery are subject to density‐dependent regulation (like larval growth rates in anuran amphibians), rebounds in population abundances may affect the ability of populations to express or use adaptive traits. Consequentially, density dependence and adaptive traits may interact in locally adapted populations to generate population dynamics that differ from ancestral populations. For example, strong density dependence may lead to smaller population sizes in locally adapted populations relative to ancestral populations. Alternatively, density‐dependent expression of adaptive traits could lead to cyclical fluctuations in population sizes of locally adapted populations. As population abundances increase and reduce the efficacy of adaptive traits, populations may undergo declines but recover once the expression of adaptive traits is no longer dampened by density‐dependent regulation. Presently, we are unaware of studies that investigate how density dependence and adaptation may interact to affect population dynamics under climate change scenarios, but similar dynamics may occur at the edges of species range expansions (Szűcs et al., 2017).

### 4.1 Niche evolution

We provide evidence that density‐dependent processes influence larval development in frog populations locally adapted to tolerate saltwater. However, when density regulated traits such as accelerated growth rates are adaptive, it is possible that traits that regulate density‐dependent processes are also under selection (Holt, 2011, 2014). For example, amphibian larvae that have adaptations leading to faster growth and development during larval development may experience selection that also favors traits that improve nutrient acquisition, competitive ability, ability to exploit new resources, or altered metabolic rates (e.g., niche evolution) (Holt, 2014). Although there is debate surrounding niche evolution (Wiens et al., 2010), evidence of niche evolution has been shown for spadefoot toads that inhabit desert environments and breed in extremely ephemeral ponds. In response to pond desiccation and food abundance, New Mexico spadefoot toads (*Scaphiopus multiplicatus*) induce a carnivorous tadpole morph capable of exploiting different resources (e.g., other tadpoles) which results in faster growth in contrast to the slower‐growing omnivorous morph (Pfennig, 1992). In our experiment, we observed very few differences between coastal and inland morphs that would suggest that density‐dependent traits have evolved in coastal populations. However, coastal frogs metamorphosed slightly longer than inland frogs even though the timing of metamorphosis was comparable. These discrepancies in length at metamorphosis could indicate that coastal tadpoles are evolving to capture or digest resources more efficiently, but this conclusion is speculative and requires further testing. A previous study comparing the gut contents of coastal and inland *H. cinerea* adults found that coastal populations had consumed a greater diversity of prey items, which is also consistent with the hypothesis that coastal frog populations may be evolving to better exploit resources (Albecker, Brantley, & McCoy, 2018).

### 4.2 Influence of dual stressors

Time to metamorphosis and exponential decay of growth rate (*α*) were most strongly affected when two stressors, salinity and high density, were combined. Specifically, the tadpoles reared in high density and 4 ppt water metamorphosed older and had higher exponential decay of growth estimates than tadpoles in the high‐density treatments reared in fresh water (Figure 4). A higher coefficient for *α* indicates that growth rates began to asymptote sooner, which could indicate that tadpoles in the 4 ppt treatment in high densities had higher metabolic costs with increasing size, greater rates of cell death, or possibly that cell growth ceased in favor of cell differentiation required for metamorphosis (Harris, 1999). Delayed metamorphosis is commonly observed in tadpoles exposed to saltwater throughout development (Karraker, Gibbs, & Vonesh, 2008; Kearney, Byrne, & Reina, 2012), but in this study, this effect is only observed in this species when combined with high densities. Delayed metamorphosis along with greater α coefficient (Equation 1—signifying rate of decay in growth through time) supports the hypothesis that increasing size may become more costly in highly crowded and osmotically stressful conditions and delay metamorphic transitions, but the mechanism underlying these patterns remains uncertain.

### 4.3 Detection of locally adapted traits

Studies on *H. cinerea* have consistently documented adaptive differences between coastal and inland populations in response to salt stress (Albecker et al., 2018; Albecker & McCoy, 2017, 2019). However, if differences among populations are masked in high density, this may have important implications for the detection and study of adaptive change in wild populations. If changes in density can mitigate the expression of adaptive traits, phenotypes produced in high‐density treatments may appear similar across environments and mask adaptive changes among populations. Therefore, care should be taken in experimental designs to control for factors that may affect the expression of targeted adaptive traits, but also to understand how those factors (like density dependence) moderate adaptive traits.

## 5 CONCLUSIONS

Density dependence has a strong influence on ecological processes and the propensity of populations to adapt to environmental change (Chevin & Lande, 2010; Richard Gomulkiewicz, Holt, & Barfield, 1999; Gurevitch, Morrison, & Hedges, 2000; May, Conway, Hassell, & Southwood, 1974; Relyea, 2000). We investigated how increased densities and salinity during larval development affect developmental traits across coastal, salt‐adapted populations and inland, salt‐naïve populations of *H. cinerea*. We found that both coastal and inland populations had reduced growth rates, greater exponential decay of growth, a smaller size at metamorphosis, took longer to reach metamorphosis, and had lower survivorship at metamorphosis in high‐density treatments. However, density and salinity affected the exponential rate of decay of growth and increased time to metamorphosis, but location of origin only affected size at metamorphosis. These findings suggest that high densities reduce survival, growth rates, size at metamorphosis, and time to metamorphosis to a greater extent than salinity or population of origin. This work also underlines the importance of understanding how density‐dependent processes mitigate adaptive responses and influence long‐term population dynamics amidst environmental change.

## CONFLICT OF INTEREST

The authors declare no conflicts of interest.

## AUTHOR CONTRIBUTIONS

MAA conceived the study, MAA, MP, MS, and JGW carried out experimentation and data collection. MAA analyzed and interpreted results. MAA, MP, MS, JGW, and MWM contributed to writing the manuscript. All authors read and approved the final manuscript.

## Data Availability

All data and code are publicly available in Dryad Digital Repository (https://doi.org/10.5061/dryad.k98sf7m3f).
